# Practical guidelines for multiple instance learning in computational pathology: how embedding choice impacts overall survival prediction

**DOI:** 10.3389/fbinf.2026.1809049

**Published:** 2026-05-20

**Authors:** Francesca Miccolis, Elisa Ficarra, Marta Lovino

**Affiliations:** 1 Department of Engineering Enzo Ferrari, University of Modena and Reggio Emilia, Modena, Italy; 2 Department of Life Sciences, University of Modena and Reggio Emilia, Modena, Italy

**Keywords:** computational pathology, foundation models, multiple instance learning, survival analysis, whole slide images

## Abstract

Whole Slide Images (WSIs) are a core data modality in computational pathology, yet their gigapixel resolution requires weakly supervised approaches such as Multiple Instance Learning (MIL) for prognostic modeling. While recent advances in representation learning have introduced domain-specific and foundation models for histopathology, it remains unclear how the choice of patch-level embedding influences survival prediction performance when combined with different MIL architectures and applied across heterogeneous cancer cohorts. Systematic evaluations addressing this gap are still limited. In this work, we present a comprehensive benchmark aimed at deriving practical guidelines for MIL-based Overall Survival (OS) prediction from WSIs. We compare four representative tile embedding strategies (ResNet50, ProvGigaPath, UNI, and CONCH) across five The Cancer Genome Atlas (TCGA) cohorts: bladder urothelial carcinoma (BLCA), breast invasive carcinoma (BRCA), colon adenocarcinoma (COAD), head and neck squamous cell carcinoma (HNSC), stomach adenocarcinoma (STAD) and one dataset from the Clinical Proteomic Tumor Analysis Consortium (CPTAC), clear cell renal cell carcinoma (ccRCC). Patch-level features are aggregated using three state-of-the-art MIL survival models: Attention-Based MIL (ABMIL), Transformer-based MIL (TransMIL), and Dual-Stream MIL (DSMIL). Furthermore, the analysis is extended to include a comparison with two state-of-the-art slide-level encoders, TITAN and the ProvGigaPath slide encoder, utilizing a Cox Proportional Hazard (CPH) model as the prediction head to benchmark patch-aggregation approaches against end-to-end slide representations. Prognostic performance is assessed using the concordance index (c-index), with interpretability evaluated through risk stratification analysis. Our results show that embedding choice has a substantial and consistent impact on OS prediction accuracy, robustness, and interpretability across cancer types, with domain-specific and foundation models outperforming conventional convolutional baselines. These findings provide evidence-based practical guidelines for designing robust and generalizable WSI-based survival prediction pipelines in computational pathology.

## 1 Introduction

Computational pathology has emerged as a key enabling discipline for quantitative cancer analysis, driven by the increasing availability of digitized Whole Slide Images (WSIs) and large-scale clinical cohorts (Madabhushi and Lee, 2016; Janowczyk and Madabhushi, 2016). WSIs capture rich morphological information at cellular and tissue levels, offering unprecedented opportunities for data-driven modeling of disease progression and patient outcome (Madabhushi and Lee, 2016; Janowczyk and Madabhushi, 2016). However, their gigapixel resolution and weakly annotated nature pose significant methodological challenges, as clinically relevant labels are typically available only at the slide or patient level (Campanella et al., 2019; Lu et al., 2021). As a result, weakly supervised learning frameworks, most notably Multiple Instance Learning (MIL), have become the *de facto* standard for extracting prognostic information from WSIs in the absence of dense annotations (Ilse et al., 2018; Campanella et al., 2019; Lu et al., 2021).

Recent advances in representation learning have substantially expanded the modeling landscape in computational pathology. Beyond conventional convolutional neural networks trained on natural images, a new generation of domain-specific and foundation models has been introduced, leveraging large-scale pretraining on histopathological data to capture richer and more transferable morphological patterns (Chen et al., 2024; Lu et al., 2024; Xu et al., 2024). These models promise improved robustness and generalization across datasets and disease types, particularly for complex downstream tasks such as survival analysis (Chen et al., 2024; Lu et al., 2024; Xu et al., 2024). In parallel, MIL-based survival models have evolved to incorporate attention mechanisms, transformer-based aggregation, and instance-level modeling paradigms, aiming to better capture intra-slide heterogeneity and handle censored clinical outcomes (Ilse et al., 2018; Shao et al., 2021; Li et al., 2021).

Despite this rapid methodological progress, existing studies on WSI-based survival prediction often differ substantially in experimental design, choice of feature extractor, cohort size, and evaluation protocol (Ghaffari Laleh et al., 2022). As a consequence, reported performance gains are difficult to compare and generalize across studies (Ghaffari Laleh et al., 2022). In particular, many works introduce novel MIL architectures or adopt powerful pre-trained embeddings without disentangling the relative contributions of representation learning and aggregation strategy (Ghaffari Laleh et al., 2022). Furthermore, evaluations are frequently restricted to a single cancer type or limited cohort sizes, hindering a systematic assessment of robustness across heterogeneous histological contexts and varying survival event rates (Ghaffari Laleh et al., 2022).

At the same time, the increasing availability of domain-specific and foundation embeddings for histopathology raises important but largely unaddressed methodological questions (Chen et al., 2024; Lu et al., 2024; Xu et al., 2024). While these representations are often assumed to improve downstream performance, systematic evidence quantifying their impact on clinically relevant endpoints such as Overall Survival (OS) remains limited, especially when considering interactions between embeddings, MIL aggregators, and cohort characteristics (Ghaffari Laleh et al., 2022). In particular, it is still unclear how embedding choice interacts with MIL survival architectures and dataset characteristics, including tumor type and cohort size, to influence prognostic accuracy, stability, and interpretability. Addressing this gap is essential for enabling reproducible and well-founded methodological choices in WSI-based prognostic modeling.

In this work, we address these limitations through a systematic, multi-cohort benchmark of embedding strategies for MIL-based OS prediction from WSIs. We evaluate four representative tile-level feature extractors spanning conventional convolutional networks, foundation models, and domain-specific architectures. Furthermore, while conventional pipelines rely on aggregating patch-level features via MIL, recent advancements have introduced slide-level architectures that learn global representations directly from the WSI. To benchmark these paradigms, our study incorporates a comparative analysis of two state-of-the-art slide-level encoders, TITAN (Ding et al., 2025) and the ProvGigaPath slide-level model (Xu et al., 2024), utilizing Cox Proportional Hazard (CPH) models to evaluate their prognostic accuracy against traditional MIL aggregation strategies. To ensure the generalizability of our findings across different institutional data sources and processing protocols, we evaluated these encoders across five cohorts from The Cancer Genome Atlas (TCGA), and one cohort from the Clinical Proteomic Tumor Analysis Consortium (CPTAC), spanning different tumours characterized by diverse histological patterns and survival profiles (Weinstein et al., 2013; Cancer Genome Atlas Network, 2012b; Cancer Genome Atlas Research Network, 2014b; Cancer Genome Atlas Network, 2012a; Cancer Genome AtlasNetwork, 2015; Cancer Genome Atlas Research Network, 2014a; Clark et al., 2019). To isolate the effect of representation learning from aggregation strategy, each embedding is combined with three widely adopted MIL survival models (Ilse et al., 2018; Shao et al., 2021; Li et al., 2021) within a unified publicly available experimental framework (Miccolis et al., 2025).

The goal of this study is to derive practical, evidence-based guidelines for the design of robust and generalizable WSI-based survival prediction pipelines in computational pathology. By jointly analyzing prognostic performance, robustness, and interpretability across embedding choices, MIL architectures, and cohort characteristics, we provide actionable insights to support informed methodological decisions in future survival analysis studies based on histopathological images.

## 2 Methods

This study is designed as a systematic, retrospective benchmark to evaluate the impact of representation learning strategies on WSI-based OS prediction. The primary objective is to isolate and quantify the contribution of both patch-level and slide-level embedding choices while controlling for aggregation strategies and evaluation protocols. To this end, we developed a unified experimental pipeline in which WSIs are either processed into patch-level embeddings for MIL-based aggregation or encoded into global representations via state-of-the-art slide-level models (TITAN and ProvGigaPath).

**FIGURE 1:**
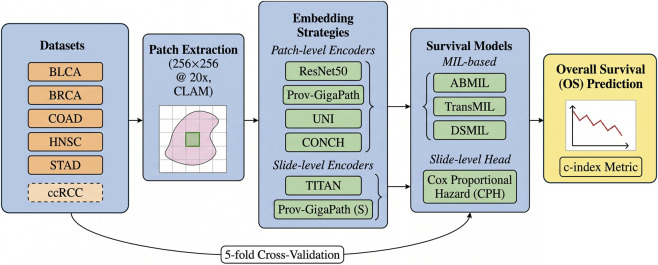
Schematic overview of the experimental workflow adopted in this study. Whole Slide Images (WSIs) are processed through tissue detection and patch extraction, followed by patch-level feature embedding using different representation learning models. Patch embeddings are aggregated using Multiple Instance Learning (MIL) survival architectures to produce patient-level risk scores, which are subsequently evaluated using survival analysis metrics.

The analysis follows a standardized workflow comprising tissue preprocessing, patch extraction, feature embedding, prognostic modeling via MIL or slide-level architectures coupled with a Cox Proportional Hazard (CPH) head, and rigorous performance evaluation across multiple TCGA and CPTAC cohorts. No model or hyperparameter tuning is performed to optimize performance on individual datasets; instead, all configurations are evaluated under comparable conditions to emphasize robustness and generalizability rather than dataset-specific optimization. An overview of the complete experimental workflow, from WSI preprocessing and patch-level feature extraction to MIL-based aggregation and survival prediction, is schematically illustrated in Figure 1.

### 2.1 Datasets and clinical endpoint

This study utilizes a total of 8,224 Whole Slide Images (WSIs) across six diverse cohorts. We selected five datasets from TCGA, namely, BLCA, BRCA, COAD, HNSC, and STAD (Weinstein et al., 2013; Cancer Genome Atlas Network, 2012b; Cancer Genome Atlas Research Network, 2014b; Cancer Genome Atlas Network, 2012a; Cancer Genome AtlasNetwork, 2015; Cancer Genome Atlas Research Network, 2014a). To evaluate external generalizability and institutional robustness, we extended the analysis to an independent multi-center cohort from CPTAC, specifically the Clear Cell Renal Cell Carcinoma (ccRCC) dataset (Clark et al., 2019). All slides consist of formaline-fixed paraffin-embedded (FFPE) tissue sections stained with Hematoxylin and Eosin (H&E).

Regarding image preprocessing, no explicit stain normalization (e.g., Macenko or Reinhard) was applied to the slides. This decision was deliberate, as recent literature on Pathology Foundation Models suggests that these models are pre-trained on massive, heterogeneous datasets precisely to be inherently robust to staining variations across different laboratories and scanners. Tissue regions were segmented at a resolution of 0.5 µm/pixel (×20 equivalent magnification), and non-overlapping patches of 256×256 pixels were extracted from the valid tissue areas.

For each cohort, patients were included if at least one diagnostic WSI and corresponding survival information were available. The clinical endpoint considered in this study is OS, defined as the time from diagnosis to death from any cause. Patients who were alive at the last follow-up were treated as censored observations. OS was chosen as a robust and clinically meaningful endpoint that is consistently available across all TCGA and CPTAC cohorts and avoids ambiguities related to cause-specific survival definitions.

A summary of cohort demographics, including the number of patients and slides, the number of censored patients, and the distribution of OS events for each cancer type, is reported in Table 1.

**TABLE 1:** Summary of cohort characteristics for each cancer type included in the study. The table reports dataset size, the number of censored patients, and the distribution of Overall Survival (OS) expressed as median and interquartile range (25th, 75th percentiles).

Cancer type	Slides	Patients	Censored	Median OS (months)	25th percentile	75th percentile
BLCA	913	412	231	13.64	7.86	21.82
BRCA	2845	1098	946	42.4	23.35	78.9
ccRCC	736	221	188	26.04	8.03	40.33
COAD	1401	460	356	13.47	5.9	35.45
HNSC	1181	474	261	14.23	8.63	26.87
STAD	1148	441	272	11.6	6.54	18.44

### 2.2 Whole slide image processing

The CLAM framework (Lu et al., 2021) was employed to extract non-overlapping patches of 256×256 pixels at 20× magnification. This magnification level was selected to provide a consistent and high-resolution basis for comparison, as ×20 (0.5 µm/pixel) represents a widely adopted standard for which pathology foundation models are designed to be highly effective. By focusing on a single high-resolution scale, we aimed to isolate the intrinsic representational quality of each encoder, avoiding the confounding architectural variables introduced by multi-scale fusion strategies. No manual annotations or region-of-interest selection were used at any stage of preprocessing, ensuring a fully weakly supervised setting. Patch-level color normalization was not applied unless explicitly stated, in order to preserve native staining variability and avoid introducing preprocessing-induced biases across cohorts.

To evaluate the effect of representation learning on survival prediction, we considered four patch-level feature extractors representing different architectural and pretraining paradigms: ResNet50, ProvGigaPath, UNI, and CONCH (He et al., 2016; Xu et al., 2024; Chen et al., 2024; Lu et al., 2024). UNI, CONCH, and ResNet50 feature extractors were executed directly within the CLAM pipeline, while ProvGigaPath embeddings were generated using the official repository implementation. ResNet50 serves as a conventional convolutional baseline commonly adopted in computational pathology (He et al., 2016). ProvGigaPath represents a large-scale foundation model pre-trained on extensive histopathology datasets (Xu et al., 2024). UNI and CONCH are domain-specific architectures designed to capture general and visual-language context-aware histopathological features, respectively (Chen et al., 2024; Lu et al., 2024). To complement the patch-level analysis, we further incorporated two state-of-the-art slide-level encoders: TITAN and the ProvGigaPath slide-level model (Ding et al., 2025; Xu et al., 2024). Unlike the tile-based extractors that require post-hoc aggregation via MIL, these models are designed to learn holistic representations of the entire WSI, enabling a direct comparison between traditional patch-aggregation paradigms and native slide-level modeling. As with the tile-level extractors, both slide encoders were used in a frozen configuration, with their global embeddings serving as inputs for a Cox Proportional Hazard (CPH) prediction head. All encoders were used in a frozen configuration without fine-tuning on downstream tasks. Patch embeddings were extracted independently for each WSI and stored for subsequent aggregation.

### 2.3 Multiple instance learning survival models

To ensure a reproducible and standardized experimental environment, we employed the OXA-MISS framework (Miccolis et al., 2025), a comprehensive and modular ecosystem designed for WSI-based survival analysis. Although natively designed for multimodal survival analysis with missing modalities, its modular architecture was leveraged here to deploy the selected unimodal aggregators (ABMIL, TransMIL, and DSMIL (Ilse et al., 2018; Shao et al., 2021; Li et al., 2021)) under strictly comparable conditions. ABMIL (Ilse et al., 2018) employs a gated attention mechanism to learn the relative importance of each patch, assigning higher weights to morphological regions most predictive of patient prognosis. DSMIL (Li et al., 2021) implements a dual-stream architecture that identifies key patches within a slide through a non-local attention strategy, facilitating instance-level modeling. TransMIL (Shao et al., 2021) utilizes a transformer-based architecture with Nyström-based self-attention to capture long-range spatial dependencies and global morphological context across the entire tissue section. This strategic selection of aggregators was intentional, as it allows for a comprehensive evaluation of the feature encoders across fundamentally different architectural philosophies: attention-weighted pooling (ABMIL), dual-stream instance-level modeling (DSMIL), and transformer-based global contextual processing (TransMIL). By spanning these diverse paradigms, we ensure that our assessment of representational quality remains robust and is not biased toward a specific aggregation logic.

Each model outputs a continuous risk score for each patient, which is optimized using a Negative Log-Likelihood (NLL) survival loss function derived from the Cox proportional hazards framework (Cox, 1972). All models were trained for 20 epochs using the AdoptAtan2 optimizer with a learning rate of $10^{-4}$ and a weight decay of $10^{-3}$ , incorporating an early stopping criterion with a patience of 5 epochs based on validation loss. Importantly, model architectures and training procedures were kept strictly consistent across all embeddings to ensure that observed performance differences could be primarily attributed to representation learning choices rather than aggregation-specific tuning.

### 2.4 Training and evaluation protocol

To ensure robust and reliable performance estimates, we employed a 5-fold cross-validation strategy for each cancer cohort. Within each fold, patients were partitioned into training, validation, and test sets, ensuring that slides from the same individual remained strictly in the same partition to prevent data leakage. The validation set was utilized for model selection and early stopping, while the independent test set was reserved for the final performance evaluation.

Model performance was evaluated using the concordance index (c-index), which measures the agreement between predicted risk scores and observed survival times while accounting for censoring. For each configuration, performance was reported as the mean and standard deviation across repeated runs. Comparative analyses were conducted both within and across cancer cohorts to assess robustness and consistency.

## 3 Results

### 3.1 Overall survival prediction performance

Table 2 summarizes the quantitative prognostic performance of all evaluated configurations, measured by the concordance index (C-index). The inclusion of both patch-level feature extractors combined with MIL aggregators and advanced slide-level encoders revealed significant variations in predictive capability across the six cancer cohorts.

**TABLE 2:** Quantitative Overall Survival (OS) prediction results. Concordance index (c-index) values (mean ± standard deviation) are reported for each combination of feature encoder and survival model across five TCGA cancer cohorts and the CPTAC ccRCC cohort.

Encoder	Model	BLCA	BRCA	ccRCC	COAD	HNSC	STAD	Mean
ResNet50	ABMIL	0.52 ± 0.08	0.55 ± 0.07	0.37 ± 0.05	0.56 ± 0.11	0.47 ± 0.05	0.63 ± 0.07	0.52 ± 0.08
	DSMIL	0.52 ± 0.09	0.58 ± 0.05	0.43 ± 0.05	0.62 ± 0.13	0.46 ± 0.11	0.63 ± 0.06	0.54 ± 0.08
	TransMIL	0.54 ± 0.05	0.61 ± 0.08	0.56 ± 0.08	0.48 ± 0.10	0.58 ± 0.06	0.53 ± 0.10	0.55 ± 0.04
ProvGigaPath	ABMIL	*0.61* ± *0.04*	0.63 ± 0.11	0.55 ± 0.09	0.51 ± 0.08	0.61 ± 0.04	0.56 ± 0.07	0.58 ± 0.04
	DSMIL	0.57 ± 0.07	*0.72* ± *0.07*	0.63 ± 0.11	0.54 ± 0.10	0.61 ± 0.04	0.57 ± 0.07	0.60 ± 0.06
	TransMIL	0.59 ± 0.05	0.66 ± 0.03	0.54 ± 0.12	0.53 ± 0.11	0.62 ± 0.06	0.65 ± 0.06	0.60 ± 0.05
UNI	ABMIL	0.58 ± 0.09	0.59 ± 0.09	0.49 ± 0.05	0.58 ± 0.07	0.57 ± 0.02	0.56 ± 0.06	0.56 ± 0.03
	DSMIL	0.56 ± 0.08	0.65 ± 0.09	0.61 ± 0.07	0.56 ± 0.11	0.63 ± 0.06	0.59 ± 0.07	0.60 ± 0.04
	TransMIL	0.58 ± 0.08	0.66 ± 0.08	0.43 ± 0.13	0.55 ± 0.06	0.63 ± 0.05	*0.66* ± *0.06*	0.58 ± 0.08
CONCH	ABMIL	0.56 ± 0.03	0.59 ± 0.12	0.54 ± 0.03	0.54 ± 0.13	0.56 ± 0.07	0.60 ± 0.05	0.57 ± 0.02
	DSMIL	0.56 ± 0.03	0.61 ± 0.09	0.61 ± 0.08	0.57 ± 0.09	*0.66* ± *0.03*	0.57 ± 0.07	0.60 ± 0.03
	TransMIL	0.53 ± 0.06	0.61 ± 0.07	0.33 ± 0.05	0.58 ± 0.09	0.62 ± 0.06	0.64 ± 0.04	0.55 ± 0.11
TITAN	CoxPH	*0.61* ± *0.06*	0.68 ± 0.06	*0.69* ± *0.08*	*0.63* ± *0.06*	0.55 ± 0.08	0.54 ± 0.06	**0.62** ± **0.06**
ProvGigaPath (S)	CoxPH	0.56 ± 0.10	0.59 ± 0.09	0.66 ± 0.08	0.61 ± 0.11	0.53 ± 0.06	0.57 ± 0.02	0.59 ± 0.04

*Italics* indicate the best result for each dataset, while bold indicates the best aggregation result overall. Higher c-index values indicate better concordance between predicted risk and observed survival times. Results are averaged across 5 cross-validation folds. For slide-level encoders (TITAN, ProvGigaPath S), a CoxPH prediction head is used.

Notably, the slide-level encoder TITAN achieved the highest overall performance across the entire experimental setup, yielding a mean C-index of **0.62** ± **0.06**. TITAN demonstrated exceptional robustness, particularly on the independent CPTAC ccRCC dataset, where it achieved a C-index of *0.69* ± *0.08*, significantly outperforming all patch-based architectures.

Among the patch-level embedding strategies, the domain-specific foundation models consistently surpassed the ImageNet-supervised baseline (ResNet50). Specifically, the combination of the ProvGigaPath encoder with the DSMIL aggregator emerged as the most robust patch-level configuration, achieving a mean C-index of 0.60 ± 0.06. This architecture performed particularly well on the BRCA (*0.72* ± *0.07*) and the ccRCC (*0.63* ± *0.11*) cohorts. Conversely, while UNI and CONCH showed competitive results on specific datasets (e.g., UNI + TransMIL achieved 0.66 on STAD), their generalization on the external ccRCC cohort was less consistent compared to ProvGigaPath and TITAN.

### 3.2 Consistency of embedding performance across cancer types

**FIGURE 2:**
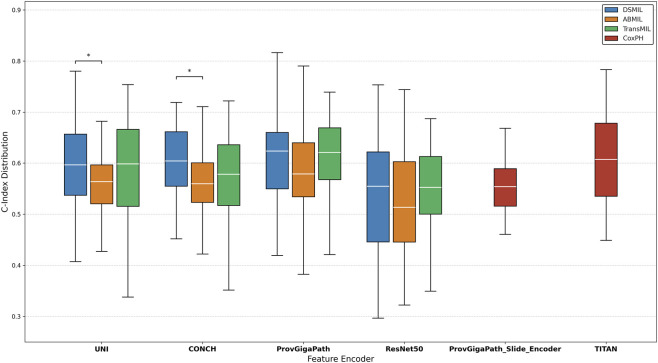
Distribution of concordance index (C-index) values summarizing Overall Survival (OS) prediction performance across all evaluated cancer cohorts. Performance is stratified by feature encoder, encompassing both patch-level models (UNI, CONCH, ProvGigaPath, ResNet50) and slide-level models (ProvGigaPath_Slide_Encoder, TITAN). For patch-level embeddings, the distributions of three Multiple Instance Learning (MIL) aggregators are shown side-by-side: Dual-Stream MIL (DSMIL) in blue, Attention-based MIL (ABMIL) in orange, and Transformer-based MIL (TransMIL) in green. For slide-level embeddings, performance is evaluated using a Cox Proportional Hazards (CoxPH) prediction head, depicted in red. Within each boxplot, the thick white horizontal line indicates the median, while the box edges and whiskers represent the interquartile ranges (Q1 to Q3) and data extremes, respectively. Asterisks (*) denote statistically significant differences between specific aggregation strategies (e.g., DSMIL vs. ABMIL for the UNI and CONCH encoders). Higher C-index values indicate better agreement between predicted risk scores and observed survival outcomes.

To investigate the robustness and stability of the representation strategies across different experimental configurations, we analyzed the distribution of survival prediction results. Figure 2 presents boxplots summarizing the C-index scores for each embedding model, aggregated over all six cancer cohorts (five TCGA and one CPTAC). The analysis encompasses both patch-level embeddings (paired with three distinct MIL aggregators) and slide-level encoders (utilizing a CoxPH head).

As shown in Figure 2, domain-specific foundation models and slide-level encoders consistently outperform the convolutional baseline. The slide-level encoder TITAN exhibits the most robust performance distribution, establishing the highest overall median and demonstrating the critical value of integrating slide-level context for survival tasks. Among the patch-level extractors, ProvGigaPath and CONCH achieve the highest median performances, with ProvGigaPath reaching the absolute peak C-index (0.72 on the BRCA cohort). UNI demonstrates remarkable stability across all MIL aggregators, suggesting it is a highly reliable, general-purpose feature extractor.

In contrast, ResNet50 exhibits the lowest median performance (∼0.55) and the lowest minimum bounds, confirming that ImageNet-supervised features are sub-optimal for capturing the complex histological patterns required for survival analysis. Furthermore, the inclusion of the CPTAC ccRCC cohort highlights that pathology-specific foundation models maintain their predictive superiority even on completely independent, highly censored external datasets.

While variability across cohorts is inherently expected due to differences in dataset size, histological heterogeneity, and survival distributions, we observe a consistent performance hierarchy. Slide-level contextualization (TITAN) and advanced pathology-specific patch embeddings (ProvGigaPath, CONCH, UNI) systematically outrank the ImageNet baseline across nearly all scenarios.

This analysis suggests that the impact of embedding choice is substantial and not restricted to a single tumor type: adopting domain-specific foundation models not only raises the average predictive accuracy but also provides a significantly higher “floor” of performance, reducing the risk of model failure on challenging clinical cohorts.

### 3.3 Impact of embedding choice on continuous risk prediction

Beyond quantitative metrics, we evaluated the clinical relevance of the embeddings by analyzing the correlation between continuous predicted risk scores and actual overall survival (OS) times. Following the rationale established in the previous section, we focused our granular analysis on the BRCA (TCGA) and ccRCC (CPTAC) cohorts using TransMIL as the fixed aggregator. This allows us to assess the stability of the prognostic signal across an internal and an independent dataset sharing analogous, highly censored statistical profiles ( >85% censored cases).

**FIGURE 3:**
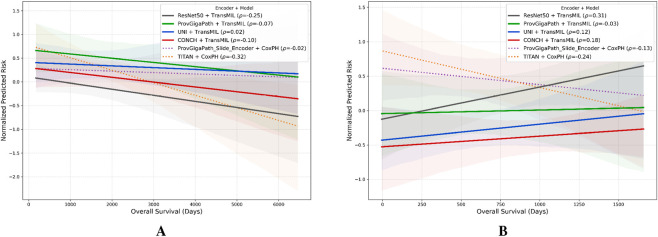
Linear fit of risk stratification analysis. Comparative regression plot of Overall Survival (OS) versus predicted risk scores using the TransMIL aggregator for tile level encoders. The BRCA **(A)** and ccRCC **(B)** cohorts were selected for comparison due to their analogous statistical profiles, characterized by high censoring rates (86% and 85%, respectively) and extended median survival times. The X-axis represents actual survival time in days, while the Y-axis represents the predicted risk score normalized to percentile ranks (0%–100%) for cross-model scale alignment. Solid colored lines denote tile-level encoders (UNI, ProvGigaPath, CONCH, ResNet50), while dashed lined denote slide-level encoders. Shaded regions represent 95% confidence intervals for the linear fit. Spearman rank correlation coefficients (ρ) for each model are reported in the legends.

Figure 3 presents a linear fit comparison of the survival trends. To enable a direct comparison across models with different output scales, raw risk scores were normalized to percentile ranks (0%–100%). To quantify the efficacy of stratification, we computed the Spearman’s rank correlation coefficient (ρ) , a non-parametric measure of rank dependence ranging from −1 to +1 . In this context, a negative ρ (closer to −1 ) indicates a correct prognostic model where higher risk scores are associated with shorter survival times. Conversely, a positive ρ implies a paradoxical relationship, and a value near 0 indicates a lack of predictive signal.

As illustrated in Figure 3, the choice of the encoder drastically alters the prognostic signal. The baseline ResNet50 (grey line) struggles to consistently stratify patients in these data-scarce regimes, often exhibiting weak correlation (ρ≈0) . This indicates that ImageNet-supervised features effectively fail to capture a meaningful prognostic gradient; higher predicted risk scores do not reliably correspond to shorter survival times.

In contrast, the domain-specific foundation models demonstrate a more robust negative correlation. The steeper downward slopes for these pathology-specific models confirm their superior ability to correctly identify high-risk patients, who exhibit significantly lower survival times (top-left of the plot) compared to low-risk patients with prolonged survival (bottom-right). This visual analysis confirms that while aggregation strategies are important, the representational quality of the patch-level features remains the primary bottleneck for effective continuous risk prediction, especially when dealing with extended survival times and sparse fatal events.

### 3.4 Risk stratification and Kaplan-Meier analysis

**FIGURE 4:**
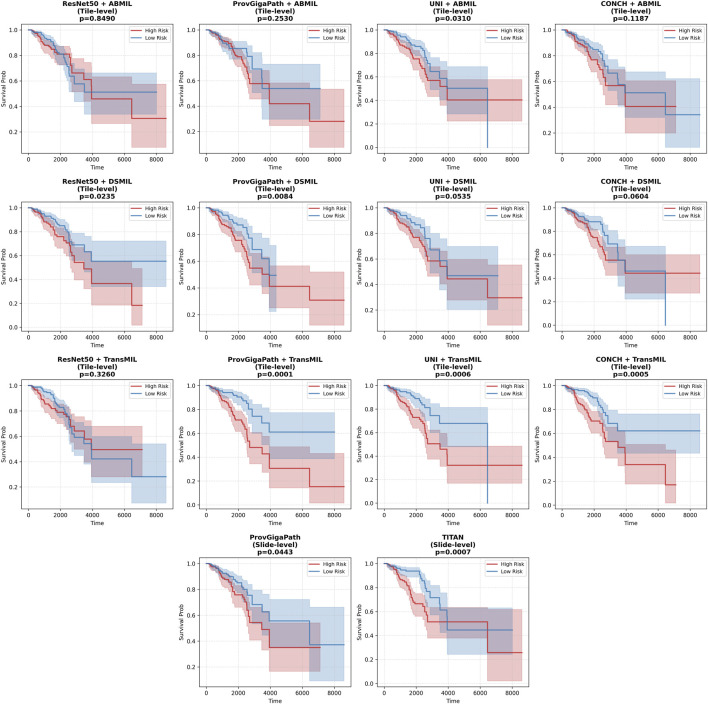
Kaplan-Meier survival analysis for the TCGA BRCA cohort. Patients were stratified into high-risk and low-risk groups based on the median predicted risk score across all evaluated embedding-aggregator configurations. The shaded regions represent the 95% confidence intervals. The p -values is calculated with log-rank test.

**FIGURE 5:**
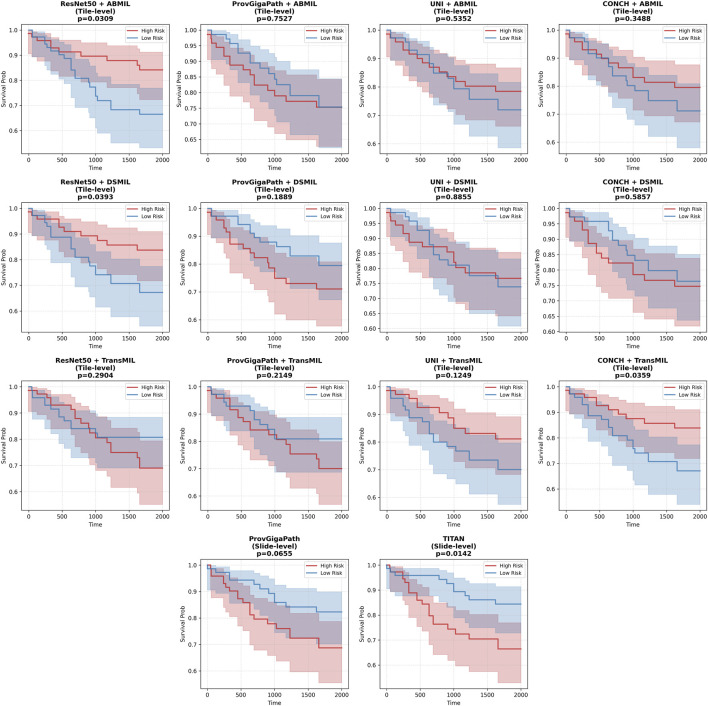
Kaplan-Meier survival analysis for the independent CPTAC ccRCC cohort. Risk stratification was performed using the median risk score thresholdacross all evaluated embedding-aggregator configurations. The shaded regions represent the 95% confidence intervals. The p -values is calculated with log-rank test.

To further evaluate the clinical relevance of the prognostic models, we performed Kaplan-Meier survival analysis. In particular, we focused on two representative datasets: the internal TCGA BRCA cohort (Figure 4) and the independent CPTAC ccRCC cohort (Figure 5). These cohorts represent the most challenging scenarios in our benchmark, featuring exceptionally high censoring rates (86% and 85%, respectively).

As shown in Figure 4, the models successfully identified populations with significantly different survival outcomes within the TCGA-BRCA dataset. Crucially, this performance was maintained on the external CPTAC-ccRCC validation set (Figure 5), confirming that the foundation model embeddings capture robust morphological signatures that translate into meaningful clinical risk categories across different cancer types and data sources.

## 4 Discussion

The primary objective of this benchmark was to disentangle the contributions of feature embeddings, ranging from classical ImageNet-supervised models to domain-specific foundation models and slide-level encoders, and MIL aggregation in WSI-based survival prediction. Our results unequivocally identify the feature extractor, and specifically the scale of context it captures, as the dominant performance factor. A consistent hierarchy emerged across cohorts: slide-level encoders (TITAN) outperformed patch-level foundation models (ProvGigaPath, UNI, CONCH), which in turn systematically outperformed the ImageNet baseline (ResNet50). Previous studies have demonstrated the utility of conventional deep learning for WSI-based risk stratification in specific cohorts (Ding et al., 2023). However, our benchmarking indicates that domain-specific foundation models systematically improve generalization, particularly on independent and highly censored datasets. This highlights a critical bottleneck: advanced aggregators like TransMIL cannot compensate for the lack of semantic signal or global context in suboptimal embeddings. Consequently, pipeline design should prioritize robust, domain-specific encoders; in resource-constrained settings, investing in superior feature extraction yields a significantly higher return than tuning complex aggregators on poor representations.

Regarding generalization, a key contribution of our expanded analysis is the validation of these models on an independent external cohort (CPTAC ccRCC). Survival prediction is notoriously susceptible to dataset bias and high censoring rates. The fact that the relative ranking of embeddings observed in the internal TCGA cohorts was maintained on the ccRCC dataset, characterized by an extreme 85% censoring rate, provides strong evidence for the cross-consortium generalizability of these models. Notably, TITAN dominated this external cohort (C-index 0.69), proving that capturing long-range spatial relationships across the entire WSI is fundamental for robust survival analysis.

Among patch-level extractors, we observed a shift in the peak performance and stability trade-off. ProvGigaPath achieved the highest performance ceiling in favorable cohorts (e.g., BRCA, C-index 0.72) and maintained strong external generalization when paired with DSMIL, emerging as the most versatile patch-based option. UNI remained a highly stable general-purpose extractor with a compact performance distribution, whereas ResNet50 failed to extract meaningful prognostic signals across almost all scenarios.

Beyond aggregate metrics, our risk stratification analysis (Figures 3–5) revealed crucial insights into clinical interpretability. Focusing on the highly censored BRCA and ccRCC cohorts, we demonstrated that foundation models and slide-level encoders produce a robust and statistically significant separation of patient trajectories. The continuous risk correlations (Figure 3) and Kaplan-Meier analyses confirm that these models capture genuine prognostic patterns, where “high risk” semantically and reliably corresponds to poorer outcomes. In contrast, the baseline ResNet50 struggled to achieve meaningful stratification in these data-scarce regimes. This suggests that future studies must validate survival models by inspecting the risk-survival gradient and stratification capability on highly censored data, rather than relying solely on the C-index.

This study has limitations inherent to its retrospective design. First, while the inclusion of the CPTAC ccRCC cohort provides a robust external validation, the observed performance hierarchy should be further verified across more diverse, multi-centric datasets to fully account for pre-analytical variations (e.g., scanners, staining protocols). Second, embeddings were evaluated in a frozen state; while necessary to isolate intrinsic representational quality, task-specific fine-tuning could potentially alter the performance dynamics, albeit at higher computational costs. Finally, potential demographic biases in TCGA and undisclosed pre-training data distributions for proprietary models warrant caution regarding generalizability to underrepresented clinical populations.

Future research should focus on three key areas: (1) investigating the exact spatial determinants (e.g., tumor microenvironment interactions): driving the slide-level superiority of models like TITAN; (2) integrating these advanced histological embeddings with clinical and genomic covariates to capture multimodal prognostic signals; and (3) exploring parameter-efficient fine-tuning strategies (e.g., adapters) to adapt foundation models to specific survival endpoints without the cost of full retraining. Establishing standardized evaluation protocols across internal and external cohorts, as demonstrated here, remains essential for measurable progress in computational pathology.

## 5 Conclusions

In this work, we conducted a systematic benchmark to evaluate how representation learning strategies, spanning patch-level embeddings and slide-level encoders, influence WSI-based Overall Survival (OS) prediction. By comparing conventional convolutional baselines against emerging domain-specific foundation models across five TCGA cohorts and an independent CPTAC ccRCC dataset, we aimed to isolate the contribution of feature extraction to prognostic modeling performance.

Our results provide compelling evidence that the representational capacity of the encoder, encompassing both semantic quality and global spatial context, is the primary driver of success in survival analysis. We observed that slide-level encoders (TITAN) and domain-specific foundation models (ProvGigaPath and UNI) systematically outperform ImageNet-supervised baselines (ResNet50) in terms of both quantitative accuracy and cross-cohort robustness. Crucially, our granular analysis of risk stratification on highly censored cohorts (BRCA and ccRCC) revealed that baseline models often fail to produce clinically meaningful risk gradients. In contrast, advanced pathology-specific models demonstrated a robust ability to reliably stratify patients into divergent prognostic trajectories, maintaining their predictive signal even under extreme data-scarce and external validation conditions.

Based on these findings, we advocate for a paradigm shift in the design of survival prediction pipelines: prioritizing the adoption of high-quality, domain-specific encoders and slide-level contextualization over the complexity of MIL aggregation architectures. While advanced aggregators (e.g., TransMIL, DSMIL) contribute to model stability and synergize well with strong embeddings, they cannot compensate for the lack of a genuine prognostic signal in suboptimal baseline features.

Overall, this study establishes a practical, evidence-based baseline for computational pathology researchers. We conclude that leveraging large-scale foundation models and slide-level architectures is not merely an option but a necessity for developing reliable, interpretable, and clinically relevant prognostic tools. Future work extending this benchmark to broader multi-centric datasets and integrating multimodal clinical variables (e.g., genomic profiles) will be essential to translate these computational guidelines into real-world clinical workflows.

## Data Availability

Publicly available datasets were analyzed in this study. This data can be found here: https://portal.gdc.cancer.gov/.
